# Eye Blink Rates and Eyelid Twitches as a Non-Invasive Measure of Stress in the Domestic Horse

**DOI:** 10.3390/ani9080562

**Published:** 2019-08-15

**Authors:** Katrina Merkies, Chloe Ready, Leanne Farkas, Abigail Hodder

**Affiliations:** 1 Department of Animal Biosciences, University of Guelph, Guelph, ON N1G 2W1, Canada; 2 Campbell Centre for the Study of Animal Welfare, University of Guelph, Guelph, ON N1G 2W1, Canada

**Keywords:** spontaneous blink rate, eyelid twitches, stress, horse, behaviour, welfare

## Abstract

**Simple Summary:**

Eye blink rate has been used as an indicator of stress in humans and, due to its non-invasive nature, could be useful to measure stress in horses. Horses exhibit both full and half blinks as well as eyelid twitches. We exposed 33 horses to stressful situations such as separation from herdmates, denied access to feed and sudden introduction of a novel object, and determined that full and half eye blinks decrease in these situations. Feed restriction was the most stressful for the horse as indicated by increased heart rate, restless behaviour and high head position. The decrease in eye blink rate during feed restriction was paralleled with an increase in eyelid twitches. There was no increase in eyelid twitches or heart rate with the other treatments indicating that the horses did not find these overly stressful, but they did focus their attention more during these situations. Observation of eye blinks and eyelid twitches can provide important information on the stress level of horses with a decrease in eye blinks and an increase in eyelid twitches in stressful environments.

**Abstract:**

Physiological changes provide indices of stress responses, however, behavioural measures may be easier to determine. Spontaneous eye blink rate has potential as a non-invasive indicator of stress. Eyelid movements, along with heart rate (HR) and behaviour, from 33 horses were evaluated over four treatments: (1) control—horse in its normal paddock environment; (2) feed restriction—feed was withheld at regular feeding time; (3) separation—horse was removed from visual contact with their paddock mates; and (4) startle test—a ball was suddenly thrown on the ground in front of the horse. HR data was collected every five s throughout each three min test. Eyelid movements and behaviours were retrospectively determined from video recordings. A generalized linear mixed model (GLIMMIX) procedure with Sidak’s multiple comparisons of least squares means demonstrated that both full blinks (16 ± 12^b^ vs. 15 ± 15^b^ vs. 13 ± 11^b^ vs. 26 ± 20^a^ full blinks/3 min ± SEM; a,b differ *p* < 0.006) and half blinks (34 ± 15^ab^ vs. 27 ± 14^bc^ vs. 25 ± 13^c^ vs. 42 ± 22^a^ half blinks/3 min ± SEM; a,b,c differ *p* < 0.0001) decreased during feed restriction, separation and the startle test compared to the control, respectively. Eyelid twitches occurred more frequently in feed restriction (*p* < 0.0001) along with an increased HR (*p* < 0.0001). This study demonstrates that spontaneous blink rate decreases while eyelid twitches increase when the horse experiences a stressful situation.

## 1. Introduction

Stress is defined as the response of an organism to environmental stimuli that threatens its internal equilibrium [1]. As a prey species, the domestic horse (*Equus caballus*) has developed adaptive fear and flight responses when faced with external stressors [2]. However, modern husbandry practices routinely subject horses to aversive stimuli such as transportation, social isolation and medical intervention. Identifying indicators of stress in the horse is fundamental for the welfare of the animal itself and the safety of the handler [3].

Various physiological measures can be used to assess stress responses in animals including heart rate and heart rate variability [4], blood or salivary cortisol [5]. However, these measures have their limitations, including the increase in stress due to the invasive nature of drawing blood, for example [6]. As a result, researchers have explored behavioural indicators to augment physiological data. For example, horses exposed to various stressors demonstrate higher head carriage [7], focused orientation of the ears [8], increased vocalizations [9] and increased mouth movements [10]. Assessing stress responses in animals appears more accurate when using a combination of both behavioural and physiological indicators [11,12]. A novel scale developed to identify stress-related behaviours subjected 32 horses to known stressful husbandry practices including the sound of electric coat clippers, social isolation and grooming procedures [13]. Moderate to high levels of stress showed an increase in oral behaviours, flared nostrils and flattened or pinned ears which correlated with an increase in heart rate (HR) and salivary cortisol [13].

However, evidence of behaviours associated with stress in horses is conflicting. Horses subjected to two stressful handling tasks—walking across a tarpaulin and walking through streamers attached to an overhead pole—displayed an increase in heart rate variability and eye temperature [14]. The time taken or willingness to complete each task was not associated with physiological indicators, showing that the horses did experience stress even when not overtly displaying stress behaviours [14]. Further, horses undergoing a hair clipping procedure, a known aversive management practice, showed compliant behaviour while displaying an increase in HR, salivary cortisol and eye temperature [15]. These studies suggest that a horse’s level of compliance and/or ability to tolerate stressors is not indicative of their level of arousal, and influences such as training may overshadow emotional responses [15].

Understanding the response of an animal to external stressors through valid behavioural indicators can be challenging and subjective, however behaviour is an easily observable and non-invasive measurement [16]. Identifying valid indicators of stress is essential to understanding the animal and ultimately improving welfare.

Eye-blink rate has been used as a non-invasive measure of arousal to predict stress levels in humans [17]. Blinking is defined as a quick movement of the eyelid that opens and closes the palpebral fissure and is composed of three different blinks: spontaneous, reflex and voluntary [18]. The levator palpebrae superioris muscle of the upper eyelid is primarily responsible for opening the eyelid, whereas the orbicularis oculi muscle encircling the palpebral fissure works to close the eyelid [19]. Upon close observation, different eyelid movements are noticeable, ranging from full blinks (complete closure of both eyelids with concomitant suppression of vision) to partial blinks (incomplete closure of the eyelids) and eyelid twitches (movement of the upper eyelid through innervation of the levator palpebrae superioris muscles with no movement of the orbicularis oculi muscles) [20]. Partial blinks have been observed in humans focused on computer terminal displays [21] and have been used as a diagnostic for dry eye disease [22]. Partials blinks have also been documented in both dogs [23] and cats [24]. Due to the large size and lateral placement of a horse’s eyes, identifying eyelid movements is easily observable. Although little investigation has been done on half blinks in horses, it has been incorporated into the Equine Facial Action Coding System (EquiFACS) as its own action unit [25].

Spontaneous blinks are uniquely different from voluntary and reflexive blinks, as they can represent a range of information processing functions spanning attention and working memory [26]. Humans subjected to stressful stimuli through social and emotional recollection tests exhibited an increase in spontaneous blink rate and a similar trend has been demonstrated in guinea pigs that are in states of emotional arousal following handling [27]. Although an increase in spontaneous blink rate has been observed in humans subjected to stressful and neurologically-demanding stimuli, spontaneous blink rate has also been found to decrease when the subject is most attentive while performing demanding tasks or exposed to stressful visual stimuli [28,29]. This suggests that humans reduce their spontaneous blink rate when perceiving visual stimuli in order to maximize the amount of information entering the nervous system; thus, increased spontaneous blink rate potentially hinders attention in humans and the ability to perceive immediate stimuli [28]. Therefore, the influence of specific stressors such as visual stimuli, emotional anxiety and/or neurological levels of arousal that initiate a fight or flight response must be considered when investigating behavioural responses in horses [17,28].

While spontaneous blink rate has been used as a non-invasive measure of stress in humans, little research has been applied to using spontaneous blink rate as a behavioural indicator in horses, and no research has differentiated between different eyelid movements. This study aims to investigate the use of spontaneous blink rate and eyelid movements as a non-invasive measure of stress in domestic horses (*Equus caballus*) in response to induced, external stressors. Based on the limited and contrasting evidence reviewed, sources of stressors including feed restriction and separation from herdmates were selected to induce social and neurological states of arousal. In comparison, the startle test was chosen to induce a stress response from visual stimuli. We hypothesized that spontaneous blink rates in horses would significantly increase during feed restriction and separation from conspecifics and decrease during the startle test in comparison to the control.

## 2. Materials and Methods

This project was approved by the University of Guelph’s Animal Care Committee (AUP #3143) in compliance with the Canadian Council on Animal Care guidelines for the use of animals in research.

### 2.1. Subjects and Housing

This study used 33 riding lesson horses (*Equus caballus*) from three different facilities in Eastern Ontario, Canada. The horses had a mean age of 11 ± 6 years, and all horses were in good health with no documented instances of digestive issues or ulcers that might impact the feed restriction treatment. As a variety of breeds were represented, they were categorized into four categories: Thoroughbred and Thoroughbred crosses (*n* = 10), warmbloods (e.g., Hanoverian; *n* = 8), stock (e.g. Quarter Horse; *n* = 7) and ponies (e.g., Welsh; *n* = 8). Before and after each treatment, the horses were housed in their usual stalls and followed their regular regimes, including being turned out with their normal herdmates and maintaining their normal exercise routines. Their diets and feeding schedules remained the same throughout the study, with the exception of the feed restriction treatment when feed was withheld.

### 2.2. Treatments

Each horse was exposed to each of the four treatments in randomized order.

**Figure 1:**
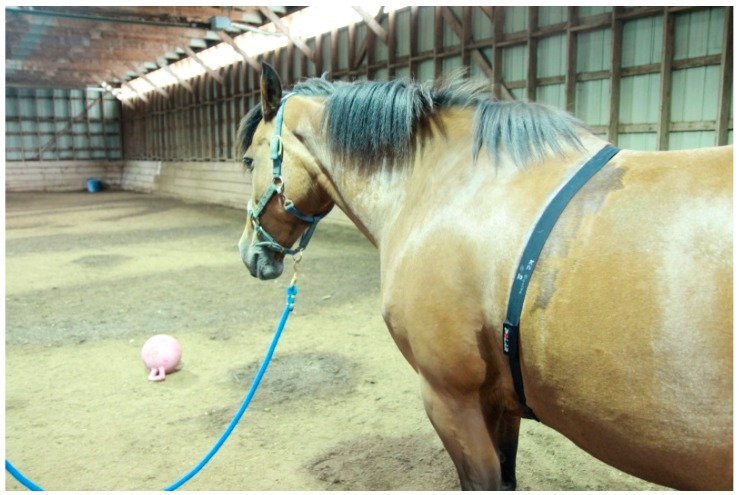
Horse with heart rate (HR) monitor (Polar RS800) during the startle test. The ball was tossed approximately 2 m in front of the horse. The handler maintained a fairly loose lead. The observer (not visible in this photo) was positioned about 3 m from the horse’s right eye.

(i) Control: the horse was observed individually for three minutes in their normal turnout environment, with the exception of the presence of the handler and observer. The horse was surrounded by, or within sight of, their paddock mates. Observations occurred during quiet times at the facility with no expectation of predictable events such as riding or feeding.

(ii) Feed restriction: the horse was tied individually in their stall during their regular afternoon feeding time, and was observed while feed was withheld for three minutes, during which time the horse watched their neighbouring conspecifics being fed.

(iii) Separation: the horse was led individually from their normal environment to an isolated testing arena. Once there, the horse was asked to stand and was observed for three minutes. There was no visual contact with their conspecifics although auditory contact was still possible. Observations occurred during quiet times at the facility with no expectation of predictable events such as riding or feeding.

(iv) Startle test: the horse was led individually from their normal environment to an isolated testing arena where they were unable to see conspecifics although auditory contact was still possible. Once there, the horse was asked to stand while a ball was thrown suddenly on the ground 2 m in front of the horse (Figure 1), and the horse was observed for three minutes. Observations occurred during quiet times at the facility with no expectation of predictable events such as riding or feeding.

### 2.3. Data Collection

All data at any one facility were collected over two or three days, and all treatments for any one horse were tested on a single day. At least 10 min prior to each treatment, each horse was outfitted with a heart rate monitor (Polar RS800, Lachine, QC, USA; Figure 1) to allow them to acclimatize to the monitors. Heart rate (HR) was collected every five s throughout each treatment.

During each treatment, a handler held the horse by a lead rope attached to the halter, and the same handler was used throughout the study. The handler held the horse in place on a fairly loose lead (1 m), with just enough contact to maintain the head relatively still without restricting movement. A single observer was used throughout the study and maintained a position about 3 m perpendicular to the right eye of the horse. The observer videotaped all treatments using a Panasonic 2MOS video camera, with focus maintained on the right eye of the horse. One individual coded all the behaviours (Table 1) retrospectively from the videos using Observer XT (Version 12.0, Noldus, Leesburg, VA, USA). All occurrences of eye blinks and eyelid twitches were tallied, while the total duration of ear movement, head movement, mouth movement and restlessness was calculated over each 3 min treatment and reported as a percentage of total time.

### 2.4. Data Analysis

The data was exported from Observer XT to Microsoft Excel 2011 (Supplementary File SBrate data.xlsx). All statistical analyses were carried out using SAS (Version 9.4, Toronto, ON), and significance was considered to be *p* < 0.05.

As there was no missing data, *n* = 33 for all analyses. Residuals were graphically inspected to determine the fit of the model, and horse HRs were log transformed since they did not achieve a normal distribution (Kolmogorov–Smirnov test, *p* < 0.01). A generalized linear mixed model procedure was used to analyse the effect of treatment on behaviour and HR, with location, age, breed and treatment as the independent variables and horse as the random factor. Sidak’s methodology was used to test multiple comparisons of least squared means for each behaviour across treatments.

## 3. Results

**Figure 2:**
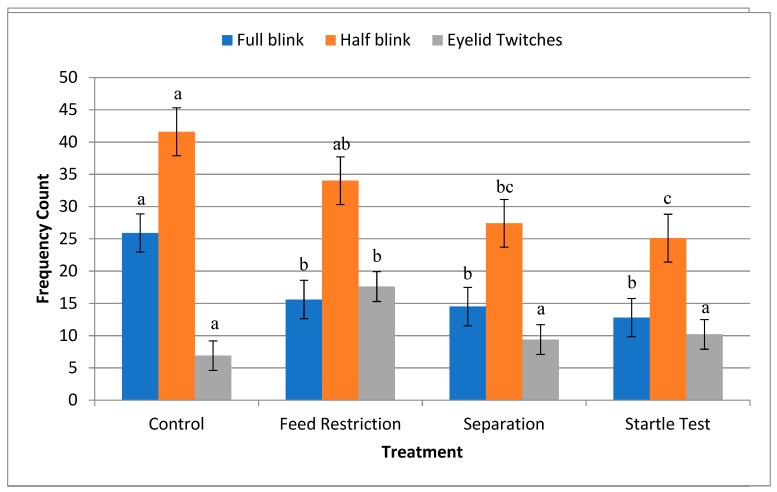
Total number of observations of full eye blinks, half blinks and eyelid twitches (±SD) in horses (*n* = 33) over a 3 min observation period during control, feed restriction, separation from conspecifics or a startle test. a,b,c differ across treatments *p* < 0.0001.

On average, horses performed full blinks 8–9 times/min in the absence of any stressors. This rate decreased to 5 blinks/min in the presence of any external stressors. Conversely, eyelid twitches increased from about 2/min in the control situation to 6/min during feed restriction. Full eye blinks occurred more often during control than during any other treatment (F(3,95) = 9.88, *p* < 0.0001; Figure 2). Half blinks occurred most often during control and feed restriction treatments, and least often during separation or startle test (F(3,95) = 10.65, *p* < 0.0001; Figure 2). Eyelid twitches were more evident during the feed restriction treatment than during any other treatment (F(3,95) = 9.46, *p* < 0.0001; Figure 2).

Horse heart rate was higher during feed restriction (44 ± 13 beats per minute (bpm)) and lower during separation (37 ± 7 bpm) and the startle test (37 ± 8 bpm) compared to the control (39 ± 8 bpm) (F(3,92) = 306.12, *p* < 0.0001). There was no effect of facility (*p* > 0.05) on the behaviours or HR.

**Figure 3:**
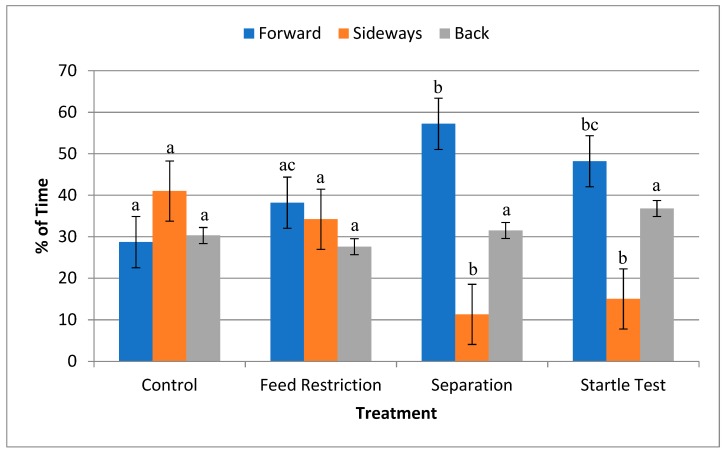
Percentage of time that horses’ (*n* = 33) ears were forward, sideways or back (±SD) over a 3 min observation period during feed restriction, separation from conspecifics, a startle test or control. a,b,c differ across treatments *p* < 0.0001.

The horses’ right ear was forward more often during separation and the startle test (F(3,95) = 8.29, *p* < 0.0001; Figure 3), whereas it was more often sideways during feed restriction and the control (F(3,95) = 22.53, *p* < 0.0001). There was no difference among treatments for the percentage of time the horses had their ears back (F(3,95) = 0.82, *p* > 0.49).

**Figure 4:**
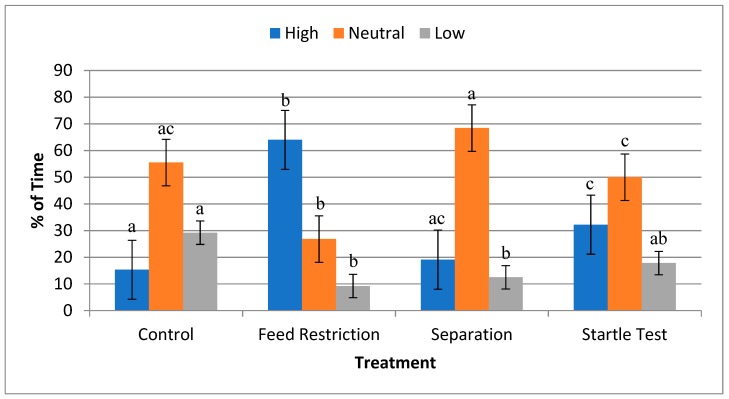
Percentage of time that horses’ (*n* = 33) heads were held high, neutral or low (±SD) over a 3 min observation period during feed restriction, separation from conspecifics, a startle test or control. a,b,c differ across treatments *p* < 0.0001.

Horses held their head raised more frequently during feed restriction (F(3,95) = 30.02, *p* < 0.0001; Figure 4) and held their head low more often during the control treatment and startle test (F(3,95) = 7.15, *p* = 0.0002).

Oral behaviours were most evident during the feed restriction, with significantly fewer during separation and the startle test (F(3,95) = 11.42, *p* < 0.0001). Horses were more often restless during feed restriction than separation or the startle test (F(3,95) = 6.78, *p* = 0.0003).

## 4. Discussion

The aim of this study was to determine changes in eyelid movement in horses during exposure to stressful stimuli. It was expected that spontaneous blink rate would increase during exposure to mental stressors such as feed restriction and separation from conspecifics and decrease during exposure to visual stimuli via the startle test. However, our results showed a decrease in both full and half blinks in response to each of the test situations.

Feed restriction was clearly the most stressful situation for the horse demonstrated by an increase in HR, restlessness, oral behaviours and percentage of time the head was held high. However, as HR increased only slightly, it may be concluded that this was only a mildly stressful situation. During separation from conspecifics and the startle test, horses had their right ear forward for more of the time, indicating focused attention in front of them that presumably maximized visual information processing. The horses may also have been attentive to sounds emanating outside of the test arena, although such sounds could come from any direction. Heart rate during these two situations was slightly decreased, showing that while attention was focused, physiologically the horses did not appear stressed. It could be that the presence of the human handler and observer may have had an appeasing effect on the horse, as Merkies et al. [7,10] demonstrated a decrease in HR when any human was present rather than the horse being completely alone.

Like full blinks, half blinks were most evident in the control environment, with the lowest frequency in the startle test where visual stimulation is important for the horse to process information about their environment. Similarly, in humans, the number of both full and partial blinks decreased when they were asked to perform a reading test requiring visual concentration [32]. Also shown in guinea pigs, eye blink rate decreased during handling compared to the control, although this study did not verify the stressful effects of handling [27]. Conversely, both full and half blinks characterized in cats increased under conditions of induced fear [24].

A recent study investigating stress responses in horses observed a decrease in mean spontaneous blink rate only during the first minute of a 10 min sham clipping procedure [33]. This decline preceded the onset of a significant increase in spontaneous blink rate for the following nine min, suggesting that the initial reduction may be characteristic of a fight or flight response, allowing the horse to visually fixate on the stimulus before responding accordingly. In our current study, blink rate was calculated as a mean over three min. It could be that a longer time frame may have noted a subsequent increase in spontaneous blink rate similar to Mott et al.’s [33] study. It would have been interesting if Mott et al. [33] had collected data on differing eyelid movements.

Our results showed an increase in eyelid twitches during feed restriction, which the horses found stressful. Conversely, separation from conspecifics and the startle test did not evoke an increase in eyelid twitches, suggesting that eyelid twitches are more prevalent during stressful situations as the horses did not appear to find these two latter situations stressful.

Research investigating horse facial expressions led to the development of the equine Facial Action Coding System (EquiFACS) for use in determining behavioural indicators of a horse’s emotional state [25]. In accordance with EquiFACS, the Equine Pain Face and Horse Grimace Scale (HGS) were created after observing changes in equine facial expressions when horses were subjected to painful procedures. These scales identified notable changes in eye expression associated with horses experiencing negative situations [31,34]. An angled eye was found to be indicative of stress by comparing the shape of the eye with the appearance of eye-white during treatments associated with negative arousal [35]. The appearance of “worry wrinkles”, defined as a contraction of the levator anguli oculi medialis muscle and the corrugator supercilii muscle, are prominent during situations involving negative arousal such as food competition, in contrast to relaxed eyes during positive stimuli such as grooming [35]. These “worry wrinkles” may be similar to eyelid twitches that appear to increase under stressful situations. As Hintze et al.’s [35] study analysed photographs, what they may have witnessed was an eyelid twitch in a static moment. Furthermore, a more recent study showed that worry wrinkles could be assessed systematically regardless of horse sex, age or breed, with the exception of angle. In this instance, thoroughbred horses displayed less contraction of the eyelid muscles than ponies, with coldbloods displaying the strongest contraction [36].

In horses, an increase in spontaneous blink rate was associated with a more anxious temperament accompanied by more movement, while a decrease in spontaneous blink rate was associated with more docile behaviour [37]. The authors proposed that these differences in temperament may be directly related to striatal dopamine levels, with anxious horses having elevated dopamine while docile horses have lower levels of dopamine. In humans, spontaneous blink rate has been positively associated with striatal dopamine production. For example, it is well known that spontaneous blink rate is decreased in humans experiencing Parkinson’s disease, an affliction that causes reduced functioning due to a decrease in dopamine production, whereas patients with schizophrenia (a hyperdopaminergic condition) exhibit increased anxiety and increased blink rate [38]. Additionally, Colzato et al. [39] demonstrated that both high and low spontaneous blink rates caused a decrease in performance in humans undergoing a start–stop task whereas those with average spontaneous blink rates performed best. These results emphasize the effect of tonic dopamine levels on spontaneous blink rate, which is an important baseline to understand changes in blink rates as a result of induced environmental stressors. It may be that a reduced spontaneous blink rate could indicate underlying pathologies such as depression, or alternatively that activity level may not necessarily reflect the level of arousal.

To our knowledge, this is the first report of the significance of eyelid twitches in horses. Blinking, and even more so eyelid twitches, are relatively easy measures to examine in horses, although the observer must be fairly close to the horse in question. Monitoring changes in blink rate, and in particular eyelid twitches, could alert the observer to changes in the level of arousal of the horse. A decrease in spontaneous blink rate concomitant with an increase in eyelid twitches may indicate a stressful situation for the horse, whereas a decrease in spontaneous blink unaccompanied by an increase in eyelid twitches may indicate an environment that is engaging but not stressful to the horse. Further research investigating specific eyelid movements in relation to level of arousal could give us insight into the emotional responses of horses. For example, in humans, facial electromyography has been successfully used to correlate facial muscle activation to positive or negative emotions. Since we cannot ask horses to self-report how they are feeling, physiological measures that differentiate between pleasant and unpleasant experiences may allow us to infer underlying emotions. Further investigation of changes in spontaneous blink rate and eyelid twitches over varying time spans is needed to identify patterns and temporal trends in response to stressful stimuli.

## 5. Conclusions

Horses exposed to stressful environments decrease their spontaneous eye blink rate and increase the frequency of eyelid twitches. However, if the environment is simply visually stimulating, eyelid twitches do not appear to increase even if eye blink rate decreases. Monitoring spontaneous blink rate is a sensitive metric of neural activity and differentiating eye blinks from eyelid twitches may provide insight on the level of arousal of the horse.

## Figures and Tables

**Table 1:** Ethogram of behaviours observed in horses (*n* = 33) during each of the four treatments—control, feed restriction, separation from conspecifics and the startle test. Adapted from [13,25,30,31].

Behaviour	Description
Eye—full blink	The right eye becomes momentarily but completely closed
Eye—half blink	The right upper lid moves toward the lower lid of the eye but does not cover the eye completely
Eyelid—twitch	Fine fibrillar movement of the skin involving the levator palpebrae superioris muscle of the upper eyelid
Ears—forward	The right ear points forward in an attentive manner
Ears—sideways	The right ear is angled to the side
Ears—back	The right ear is turned backward
Head—above withers	The right eye level goes above the height of the withers
Head—even with withers	The right eye is even with the height of the withers
Head—below withers	The right eye level drops below the height of the withers
Oral behaviour	The lips are in motion, either with mouth shut, with the tongue licking or coming out of the mouth, or chewing
Restlessness	Any movement made by the legs, including movement that causes the horse to move out of view of the camera

## Supplementary Materials

The following are available online at https://www.mdpi.com/2076-2615/9/8/562/s1.
